# Nanobodies as potential tools for microbiological testing of live biotherapeutic products

**DOI:** 10.1186/s13568-023-01659-z

**Published:** 2024-01-20

**Authors:** Robert J. Dorosky, Jeremy E. Schreier, Stephanie L. Lola, Rosa L. Sava, Michael P. Coryell, Adovi Akue, Mark KuKuruga, Paul E. Carlson, Sheila M. Dreher-Lesnick, Scott Stibitz

**Affiliations:** 1 Office of Vaccines Research and Review, Division of Bacterial, Parasitic and Allergenic Products, Center for Biologics Evaluation and Research, U.S. Food and Drug Administration, Silver Spring, MD USA; 2 Department of Marine Sciences, University of Georgia, Athens, GA USA

**Keywords:** Nanobody, Live biotherapeutic products, Biologics, *Lactobacillus*, Potency assays

## Abstract

Nanobodies are highly specific binding domains derived from naturally occurring single chain camelid antibodies. Live biotherapeutic products (LBPs) are biological products containing preparations of live organisms, such as *Lactobacillus*, that are intended for use as drugs, i.e. to address a specific disease or condition. Demonstrating potency of multi-strain LBPs can be challenging. The approach investigated here is to use strain-specific nanobody reagents in LBP potency assays. Llamas were immunized with radiation-killed *Lactobacillus jensenii* or *L. crispatus* whole cell preparations. A nanobody phage-display library was constructed and panned against bacterial preparations to identify nanobodies specific for each species. Nanobody-encoding DNA sequences were subcloned and the nanobodies were expressed, purified, and characterized. Colony immunoblots and flow cytometry showed that binding by Lj75 and Lj94 nanobodies were limited to a subset of *L. jensenii* strains while binding by Lc38 and Lc58 nanobodies were limited to *L. crispatus* strains. Mass spectrometry was used to demonstrate that Lj75 specifically bound a peptidase of *L. jensenii*, and that Lc58 bound an S-layer protein of *L. crispatus*. The utility of fluorescent nanobodies in evaluating multi-strain LBP potency assays was assessed by evaluating a *L. crispatus* and *L. jensenii* mixture by fluorescence microscopy, flow cytometry, and colony immunoblots. Our results showed that the fluorescent nanobody labelling enabled differentiation and quantitation of the strains in mixture by these methods. Development of these nanobody reagents represents a potential advance in LBP testing, informing the advancement of future LBP potency assays and, thereby, facilitation of clinical investigation of LBPs.

**Supplementary Information:**

The online version contains supplementary material available at 10.1186/s13568-023-01659-z.

## Key points

- Lc38, Lc58, Lj75, and Lj94 exhibit sub-species-level binding specificity.
- Lj75 and Lc58 bind strain-specific epitopes on cell surface-exposed proteins.
- Fluorescent Lj75 and Lc58 allow enumeration of *Lactobacillus* strains in mixture.

**Supplementary Information:**

The online version contains supplementary material available at 10.1186/s13568-023-01659-z.

## Introduction

Nanobodies are recombinant, single-domain antigen binding fragments derived from naturally occurring single chain, camelid heavy chain-only antibodies (HcAbs) (De Meyer et al. 2014; Muyldermans 2001) and exist as small, stable, monomeric entities. This aspect greatly facilitates cloning of nanobody fragments, phage display library generation, genetic manipulation, expression in bacterial systems, and solubility (Muyldermans 2001). Given these properties, nanobodies are proven tools for use in fluorescent subcellular labelling of proteins of interest, immunoprecipitation, and as adjuncts to protein crystallization (De Meyer et al. 2014; Muyldermans 2001, 2013; Rothbauer et al. 2006, 2008). Moreover, nanobodies have recently been generated and engineered for therapeutic and diagnostic applications (Abbady et al. 2012; De Meyer et al. 2014; De Tavernier et al. 2016; Pymm et al. 2021). For example, a diagnostic assay using nanobodies to detect *Listeria* in milk was recently described (Tu et al. 2016). The highly specific binding characteristics of nanobodies in conjunction with the ease with which they are manipulated in the lab makes them amenable to fluorescent tagging and makes them potentially useful as probes to evaluate the microbiological composition and characteristics of multi-strain live-biotherapeutic products (LBPs).

LBPs are biological products that consist of live organisms, such as bacteria, that are intended to prevent, treat, or cure a disease or condition of human beings, and are not vaccines (FDA 2016). The ongoing microbiome revolution has resulted in an explosion of information about the role of the human microbiome in health and disease and has fueled interest in evaluating LBPs in controlled clinical trials. The FDA considers LBPs as investigational drugs and they are regulated as such. One aspect of this regulation is that microbiological testing is needed to ensure participant safety and manufacturing consistency (Dreher-Lesnick et al. 2017).

One issue often encountered in LBP product development is developing adequate product potency assays. These assays should ideally be able to specifically identify and enumerate viable cells of each strain in a multi-strain product. The number of viable microorganisms in an LBP is a key parameter used to assure that, when tested in a clinical study or used as a licensed product, it is given at the intended dose. It is also used as an important indicator of manufacturing consistency and is a primary variable measured in assessing the stability of a product. LBP potency is typically a measurement of total viable cells of each strain in a product, commonly determined by plating products to determine colony forming units (CFU) per dose. In the case of multi-strain products, it may be difficult to distinguish between different strains on the basis of colony morphology or behavior on indicator media, particularly in the absence of identified selective growth conditions for each individual strain. Here we address these challenges through the development of fluorescent nanobody probes that specifically bind individual strains in a bacterial mixture, allowing both an assessment of viability and individual strain enumeration using colony immunoblots or flow cytometry.

*Lactobacillus* species are naturally occurring members of the human microbiome that are considered to potentially confer health benefits to their hosts, as reflected by the presence of lactobacilli in marketed probiotic formulations and investigation of their purported health benefits in clinical trials (clinicaltrials.gov). Macromolecules present on the surface of *Lactobacillus* cells are important elements at the interface between bacteria and their environment, which is the human host in the context of the human microbiome (Lebeer et al. 2008, 2010; van Baarlen et al. 2013). A key factor influencing the cell surface constituents of many *Lactobacillus* species is the ability to cover their cells in surface layer protein arrays or S-layers (Johnson et al. 2016). In this study, we exploited the differences in S-layer- and non-S-layer-producing *Lactobacillus* cell surfaces to develop reagents that can be used to identify *Lactobacillus* strains by colony immunoblot and flow cytometry. We developed fluorescent nanobody probes against an S-layer-producing *L. crispatus* strain as well as a non-S-layer-producing *L. jensenii* strain. These nanobodies were used to explore two objectives: (1) identify unique surface antigens accessible during different growth conditions so that these proteins can be exploited for development of future specific binding reagents, and (2) use the fluorescent nanobodies to identify and enumerate the viable cells of two *Lactobacillus* strains in a mixture, after growth on solid media by colony immunoblotting or directly, by flow cytometry. This study demonstrates that fluorescent nanobodies can be useful reagents for characterizing and enumerating *Lactobacillus* species and identifying types of surface antigens that may be targeted in the future, and in other organisms, to generate additional reagents for the development of LBP potency assays.

## Materials and methods

### Bacterial strains and plasmids

The strains and plasmids used in this study are described in Additional file 1: Table S1. All *Lactobacillus* strains were grown in De Man, Rogosa and Sharpe (MRS) agar (Oxoid) under anaerobic conditions at 37 °C (Whitley workstations DG250; Microbiology International) or statically at 37 °C in MRS broth (Oxoid) under atmospheric oxygen conditions. *Escherichia coli* strains were grown on Luria-Bertani (LB) agar or in LB broth at 37 °C. When appropriate, *E. coli* BL21 and *E. coli* SHuffle (NEB) strains were grown in LB medium supplemented with 100 µg/ml Ampicillin (Amp).

### Camelid nanobody generation, selection, and synthesis

The camelid single domain antibodies were isolated and produced by GenScript USA. Llamas were immunized with radiation-killed bacterial preparations containing 4 × 10^8^ CFU/ml of *L. crispatus* 33820, *L. crispatus* 33197, or *L. jensenii* JV-V16. These cells and the control antigens used in screening were grown in MRS broth until the cultures reached approximately 10^10^ CFU/ml and the cells were centrifuged (4000×*g*, 10 min), and resuspended in PBS. The cells were diluted to approximately 4 × 10^8^ CFU/ml, irradiated with a Gammacell 1000 Elite for 6 h. The llamas were immunized with the *L. crispatus* antigen (1:1 strains 33820 and 33197) or *L. jensenii* JV-V16 antigen by GenScript. Two different *L. crispatus* strains were used to immunize llamas so that nanobodies with a broader range of *L. crispatus* binding would be generated. Phage display libraries for *L. crispatus* and *L. jensenii* specific nanobody fragments was generated by GenScript. The phage display libraries were panned against *L. crispatus* or *L. jensenii* target cells and binding clones were identified by phage ELISA using a HRP/Anti-monoclonal antibody. Nanobody fragments that bound the target cells (*L. crispatus* or *L. jensenii*) but not other lactobacilli were identified using the fast screening for expression, biophysical properties, and affinity (FASEBA) screening method (GenScript). Lc58 and Lc38 encoding nanobody fragments were chosen for characterization because they exhibited binding to the target *L. crispatus* antigen (strains 33820 and 33197), but not to the *L. rhamnosus* (D), *L. paracasei* (V), *L. plantarum* (V), *L. acidophilus* (V), or *L. jensenii* JV-V16 control antigens (Additional file 1: Fig. S1A). Likewise, Lj75 and Lj94 were chosen for characterization because they had binding to the target *L. jensenii* JV-V16 antigen, but not to the *L. gasseri* JV-V03 and *L. reuteri* CF48-3 A control antigens (Additional file 1: Fig. S1B). The selected nanobodies were sequenced and the nucleotide sequences encoding Lj75, Lj94, Lc58, and Lc38 have been assigned NCBI accession numbers OR295635, OR295636, OR295637, and OR295638, respectively.

### Cloning and in trans expression of nanobody-fluorescent proteins and putative nanobody targets

The expression constructs encoding the nanobody, nanobody-fluorescent protein fusions, and the Lj75 target candidates were generated by GenScript USA. The nanobody fluorescent protein constructs were designed as follows: the nanobody encoding sequence is upstream and in-frame of a sequence encoding a (G_4_S)_3_ flexible linker sequence that is upstream and in frame with a fluorescent protein coding sequence. GenScript generated synthetic DNA sequences where Lc58 was genetically fused to the TagRFP coding sequence whereas Lj75 was fused to TagBFP and TagGFP coding sequences (Drobizhev et al. 2011; Subach et al. 2008). For the nanobody-fluorescent protein fusion expression and candidate constructs (accession numbers in Table 2), codon optimized synthetic DNA fragments encoding the constructs were cloned into pET22b (+) between the XbaI and XhoI sites. The Lc58 candidate targets were amplified from the *L. crispatus* 125-2-CHN genome using primer pairs SDL169-194, SDL170-195, and SDL172-196 and cloned into pET22b (+) using XbaI and XhoI sites. The nanobody sequences were inserted upstream of and in frame with a HisTag sequence and the nanobody target candidates were inserted upstream and in frame with a FLAG tag. The nucleotide sequences encoding Lc58-TagRFP, Lj75-TagBFP, Lj75-TagGFP, EEU18441.1, EEU19392.1, EEU18637.1, EFH305441.1, EFH30000.1, and EEX23860.1 have been assigned NCBI accession numbers OR295639, OR295640, OR295641, OR362328, OR362329, OR362330, OR362331, OR362332, and OR362333, respectively. For the fluorescent nanobody constructs, the fluorescent nanobody encoding plasmids were transformed into *E. coli* Shuffle and the nanobody candidate constructs were transformed into *E. coli* BL21.

The *E. coli* BL21 (DE3) or *E. coli* SHuffle strain carrying the expression construct of interest was grown overnight with agitation (200 RPM) at 37 °C in LB broth supplemented with appropriate antibiotics. Cells were sub-cultured into fresh LB broth supplemented with appropriate antibiotics and incubated with shaking (200 RPM) at 37 °C for 2 h or until the culture reached an OD_600_ of 0.6. Protein expression was then induced by the addition of 1 mM IPTG, and cells were grown overnight with agitation (200 RPM) at 30 °C (nanobody targets) or 18 °C (fluorescent nanobodies). Cells were pelleted (4000×*g* at 4 °C for 10 min) and the pellets were stored at − 80 °C until use.

### Purification of nanobody recombinant proteins

His-tagged nanobodies Lj94, Lj75, Lc38, and Lc58, purified by Ni column affinity chromatography were obtained from GenScript USA. Fluorescently tagged nanobodies (Nb-FP) were purified according to the Talon Superflow manufacturer’s instructions (GE Healthcare). Nb-FP expression was induced in cultures as described above and cell pellets were stored at − 20 °C until use. The pellet was resuspended in binding buffer (50 mM Sorenson Phosphate Buffer, 150 mM NaCl, 10 mM imidazole, pH 7.5) supplemented with DNase1, lysozyme (0.5 mg/ml). Cell debris was removed by centrifugation (4000×*g*, 10 min, 4 °C) and the supernatant was passed through a 0.45 μm filter (Millipore). Talon Superflow resin was prepared according to the manufacturer’s instructions and proteins were eluted in elution buffer (50 mM Sorenson Phosphate Buffer, 150 mM NaCl, 150 mM imidazole pH 7.5). Preparations were buffer-exchanged using Amicon Ultra centrifugal filters—10K (Millipore) and resuspended in PBS buffer (pH 7.2). A BCA Protein Assay Kit (Pierce) was used to quantify total protein, following the manufacturer’s instructions. Proteins were analyzed by SDS PAGE for purity (Additional file 1: Fig. S2).

### Colony immunoblot assays

*Lactobacillus* strains were streaked onto MRS agar and grown for 48 h under anaerobic conditions at 37 °C. Colonies from each strain were picked and then patched onto a fresh MRS agar plate fitted with a 50-square grid. After 48 h, an 82-mm nitrocellulose membrane (Whatman Protran BA85) was laid onto the plates, then lifted, along with adherent bacterial growth and placed into PBST buffer (1× PBS with 0.05% Tween-20, pH 7.4). Blots were washed 3× in PBST at room temperature (RT). The blot was blocked in PBST + 5% BSA and washed 3× in PBST. Then they were incubated with 2 µg/ml purified nanobody proteins in PBST + 5% BSA for 1 h as described above. Finally, blots were washed in PBST and then incubated with a 1:5000 dilution of HisProbe HRP-conjugated antibody (Thermo Scientific) for 1 h at RT with gentle shaking (50 RPM). After final washing, blots were incubated with ECL Plus per manufacturer’s instructions (Cytiva). For the fluorescent nanobody assay, the blots were incubated with Lc58-RFP (50 µg/ml) and Lj75GFP (25 µg/ml), washed, and then imaged. The blots were imaged with a Gbox Chemi XX6 Imager (Syngene) using the ECL western blot program for non-fluorescent nanobody binding and Texas Red and TurboGFP filters to detect fluorescent nanobody binding. Image analysis was conducted with GeneTools Software.

### Flow cytometry binding assays

*Lactobacillus* strains were grown statically overnight at 37 °C in MRS broth. Cultures were sub-cultured to OD_600_ 0.03 in fresh MRS broth and grown statically for 24 h at 37 °C. For growth phase binding assays, samples were also collected during exponential and stationary phases. Cells were washed via centrifugation (4300×*g*, 10 min) and resuspended in PBS (pH 7.2) to OD_600_ 0.8. Cell solutions were mixed with purified Lc38 (10 µg/ml), Lj94 (10 µg/ml), Lc58TagRFP (10 µg/ml), or Lj75TagBFP (50 µg/ml) in a 96-well plate and incubated with gentle shaking at room temperature for 1 h. For the multiplexing experiments, *L. jensenii* 115-3-CHN and *L. crispatus* 33820 mixtures were incubated with Lc58TagRFP (10 µg/ml) and Lj75TagBFP (50 µg/ml). After incubation, samples were washed 3× with PBS by centrifugation (4300×*g*, 15 min) and resuspended in PBS. The Lj75TagBFP and Lc58TagRFP treated samples were ready for flow cytometry analysis after washing. The Lc38 and Lj94 treated samples were treated with 5 µg/ml of anti-his Dylight 488 (Invitrogen), incubated for 1 h at room temperature, and washed (3×) prior to flow cytometry analysis. Prior to flow cytometry analysis of multiplex mixtures, a sample was collected for CFU determination, and the remaining mixture was treated with a live/dead stain (SYTOX Green Ready Flow Reagent, Thermo Fisher Scientific). CountBright Absolute Counting beads (Invitrogen) were then added to samples for the enumeration experiments according to the manufacturer’s instructions. Flow cytometry plates were analyzed on a BD LSR Fortessa X-20 Flow Cytometer using a 0.5 µl/s sample flow rate, and 15 µl aliquots of each sample. At least 10,000 events were acquired for each sample. Data analysis was performed using FlowJo Software (FlowJo). The viable cells/ml of the original bacterial mixture were calculated following the CountBright Absolute Counting Beads manufacturer’s instructions. Flow cytometry viable cell enumeration and relative strain abundance results were compared to CFU derived by plating dilutions of the original bacterial mixture.

### Western blots

Stationary phase *L. crispatus* 125-2-CHN, *L. jensenii* JV-V16, and *L. jensenii* 115-3-CHN cultures were pelleted by centrifugation, washed with PBS, and the pellets were stored at − 80 °C. *E. coli* strains expressing recombinant candidate nanobody target proteins were grown in LB broth, induced for expression with 1 mM IPTG, incubated for an additional 4 h, and pelleted at 4000×*g*, 4 °C, 15 min. *Lactobacillus* and nanobody target pellets were resuspended in lysis buffer (PBS, 1× BPER, DNaseI, lysozyme) and incubated at 37 °C for 1 h with shaking. Cellular debris was removed by centrifugation (4000×*g*, 4 °C, 15 min) and the supernatant was passed through a 0.45 μm filter (Millipore). Samples were run on an SDS PAGE gel and proteins were transferred to PVDF membranes and probed with 50 µg/ml of Lc58 or Lj75 in PBST + 3% BSA followed by HisProbe (1:5000) in PBST + 3% BSA. ECL Plus reagent (Pierce) was used to develop the blot. To confirm expression of the FLAG-tagged proteins (nanobody target preparations only), a blot was probed with a primary M2 αFLAG antibody (Sigma Aldrich) (1:5000), a secondary anti-mouse goat antibody (Santa Cruz Biotechnology) (1:5000) and developed with ECL Plus.

### His-trap of nanobody bound proteins

To identify the antigen recognized by Lc58 and Lj75, a His-trap protocol was employed (Thermo Fisher Scientific Resin Batch protocol and Diana Oram *pers. comm.*). *L. jensenii* 25258, *L. crispatus* 125-2-CHN, *L. jensenii* JV-V16, and *L. jensenii* 115-3-CHN cultures were pelleted and the supernatant was discarded, pellets were washed with PBS, and re-suspended in Ni-NTA equilibration buffer (10 mM imidazole). Cells were lysed via sonication (6, 15 s pulses with intervening 10 s rest periods) and debris was removed by centrifugation (4000×*g*, 20 min, 4 °C) and passage through a 0.45 μm filter (Millipore). The filtered supernatant was combined with one of the nanobodies (Lc58 or Lj75) in Ni-NTA equilibration buffer or Ni-NTA equilibration buffer alone and incubated at 37 °C for 1 h. Ni-NTA resin was prepared following the manufacturer’s instructions (Thermo Fisher). After incubation, the samples were mixed with Ni-NTA resin (2:1 volume of lysate to resin) and agitated with gentle rocking for 1 h at RT. The resin was washed 5× with Ni-NTA wash buffer (25 mM imidazole; 700×*g*, 2 min, 4 °C). After washing, loading dye (NuPAGE, Invitrogen) was added to each sample, incubated at 98 °C for 10 min to release protein from the resin, and the samples were run on a SDS PAGE gel. A band of the appropriate size was excised from the gel and analyzed by mass spectrometry (MS). The presence of the target protein was confirmed by Western blot on a duplicate gel by the presence of a band at the location corresponding to where the gel band was excised.

### Mass spectrometry analysis of nanobody bound proteins

MS sample preparation, analysis, and database search were performed by the Facility for Biotechnology Resources (CBER, FDA) on a Nano Flow LC–MS using Fusion Orbitrap (Thermo Scientific). Raw data files were analyzed against the genomic sequences of the corresponding strains using Protein Discovery (version 1.4) with standard search and filtering parameters. The SignalP 4.1 Server was utilized to identify which of the enriched proteins were predicted to be secreted. Proteins that were enriched with two or more unique peptides in the nanobody pull-down samples compared to the negative controls were analyzed further. The *L. jensenii* 115-3-CHN candidate protein (EEX23860.1) was identified by searching the *L. jensenii* 115-3-CHN protein database using the AA sequence of *L. jensenii* JV-V16 candidate, EFH30000.1, as a query. The start codon of EEX23860.1 had been incorrectly annotated in the *L. jensenii* 115-3-CHN genome resulting in a predicted protein sequence that was only 38.2 kDa and lacked a signal peptide (Additional file 1: Fig. S3). Using the correct start codon, the EEX23860.1_EX amino acid sequence is predicted to possess a signal peptide and to have an 80.6 kDa molecular weight (Additional file 1: Fig. S3).

### Fluorescence microscopy

*Lactobacillus* cell preparations were prepared as described above (flow cytometry assays) and the cell preparation was mixed with 100 µl of Lc58RFP (50 µg/ml) or Lj75BFP (50 µg/ml) or both and incubated for 1 h as described above. Samples were washed 3× by centrifugation (4300×*g*, 15 min) and resuspended in PBS. After the final centrifugation step, the cell pellet was resuspended in 50 µl PBS and 2 µl of sample was pipetted onto a 1% agarose pad and allowed to air dry. The sample was visualized with a Nikon eclipse Ci fluorescence microscope with AT-TRITC and AT-EGFP filters. Image analysis was conducted with NIS Elements BR software.

### Colony identification using MALDI-TOF mass spectrometry

The Bruker MALDI Biotyper (MBT) Smart system (Bruker Daltonics, Billerica MA) was utilized to differentiate *L. crispatus* 33820 and *L. jensenii* 115-3-CHN colonies grown on MRS agar plates as previously described (Coryell et al. 2023). Briefly, *L. crispatus* 33820 and *L. jensenii* 115-3-CHN were added to the Main Spectra Profile (MSP) library according to the manufacturer’s instructions. The plate was processed on the Bruker MALDI Biotyper System per the manufacturer’s instructions and the data were analyzed with FlexAnalysis software. A similar protocol was used to identify *L. jensenii* and *L. crispatus* colonies that grew on MRS plates. Briefly, single colonies were transferred to disposable MALDI target chips, spotted with 1 µl of 70% formic acid, and allowed to dry. The dried spots were overlaid with 1 µl of HCCA matrix solution and allowed to dry. Finally, the plate was processed on the Bruker MALDI Biotyper System using Bruker flexControl (v 3.4) and MBT Compass (v 4.1) software for instrument operation and identification, respectively.

## Results

### Binding specificity of Lc58, Lc38, Lj94, and Lj75 nanobodies

**Fig. 1:**
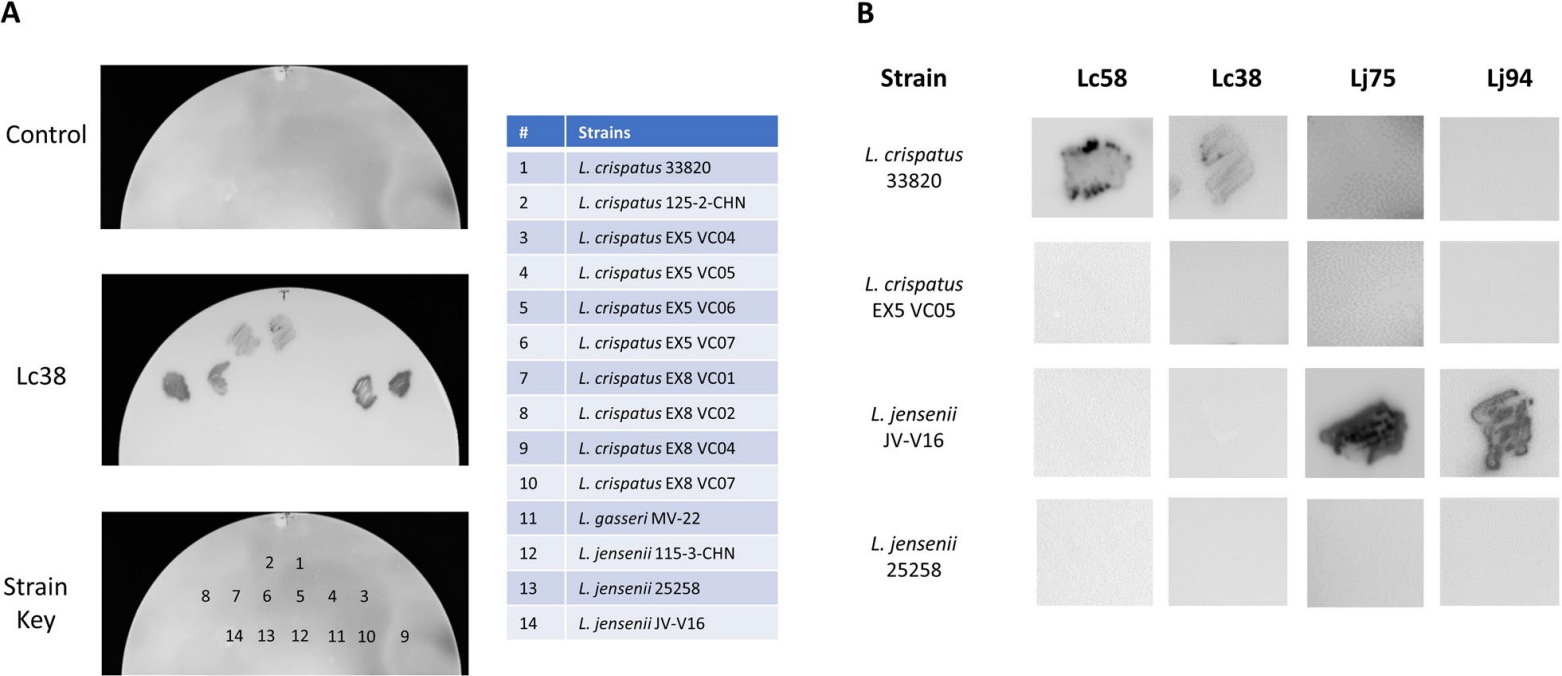
Binding specificity of nanobodies by colony immunoblots. *Lactobacillus* strains were patched or spotted onto MRS agar and transferred to nitrocellulose filters, which were probed with Lj94, Lj75, Lc38, or Lc58 as described in “Materials and methods”. **A** Blot of *L. crispatus* strains probed by buffer control (top) or Lc38 (middle) and a strain key (bottom) with corresponding table (right) identifying the location of each *L. crispatus* strain on the blot. **B** Summary results of immunoblot experiments with Lc58, Lc38, Lj75, and Lj94

Nanobodies Lc58 and Lc38 were isolated from llamas immunized with an radiation-killed preparation of *L. crispatus* strains 33820 and 33197 whereas nanobodies Lj75 and Lj94 were isolated from animals immunized with radiation-killed preparations of *L. jensenii* JV-V16 (details in “Materials and methods”). These nanobodies were chosen for further characterization because they exhibited binding to the antigen strains (i.e. *L. crispatus* or *L. jensenii*) but not to control antigens composed of other radiation-killed lactobacilli strains (“Materials and methods” and Additional file 1: Fig. S1). Enumerating and identifying colonies formed after plating on solid media or direct measurement of viable cells in mixture by flow cytometry are two potential approaches to measuring LBP potency with specific labelling reagents. To assess the utility of the nanobodies Lc58, Lc38, Lj94, and Lj75 in this regard, their ability to bind *Lactobacillus* strains was assessed by both methods. For the colony immunoblot assay, *Lactobacillus* colonies were transferred to a nitrocellulose filter and probed with the nanobodies and a secondary anti-His HRP conjugated antibody. HRP activity observed on *L. crispatus* 33820 patches probed with Lc38 and Lc58 and *L. jensenii* JV-V16 patches probed by Lj75 and Lj94 indicated that these strains were bound by these nanobodies (Fig. 1A, B). Nanobodies Lj75, Lj94, Lc58, and Lc38 bound to the strains against which they were raised (Fig. 1B; Table 1) and these nanobodies exhibited binding to some, but not all, additional strains of the same species. They did not bind to any examples of species against which they were not raised (Table 1). For example, Lj75 and Lj94 bound a subset of the *L. jensenii* strains and only *L. jensenii* JV-V16, respectively. These nanobodies did not bind any of the *L. crispatus*, or *L. gasseri* strains tested (Table 1). These results indicate that the antigens targeted by Lc38, Lc58, and Lj75, Lj94 are detectable by colony immunoblotting.

**Table 1:** Immunoblot binding specificity of nanobodies Lc58 and Lj75

Strain	Lc58	Lc38	Lj75	Lj94
*L. crispatus* 125-2-CHN	+	+	−	−
*L. crispatus* EX533959 VC04	−	−	−	−
*L. crispatus* EX533959 VC05	−	−	−	−
*L. crispatus* EX533959 VC06	−	−	−	−
*L. crispatus* EX533959 VC07	−	−	−	−
*L. crispatus* EX849587 VC01	+	+	−	−
*L. crispatus* EX849587 VC02	+	+	−	−
*L. crispatus* EX849587 VC04	+	+	−	−
*L. crispatus* EX849587 VC07	+	+	−	−
*L. crispatus* 33820*	+	+	−	−
*L. crispatus* 33197*	+	+	−	−
*L. gasseri* MV-22	−	−	−	−
*L. jensenii* 115-3-CHN	−	−	+	−
*L. jensenii* JV-V16**	−	−	+	+
*L. jensenii* 25258	−	−	−	−

*nt* not tested

*Indicates strains used to generate Lc58

**Indicates strain used to generate Lj75

**Fig. 2:**
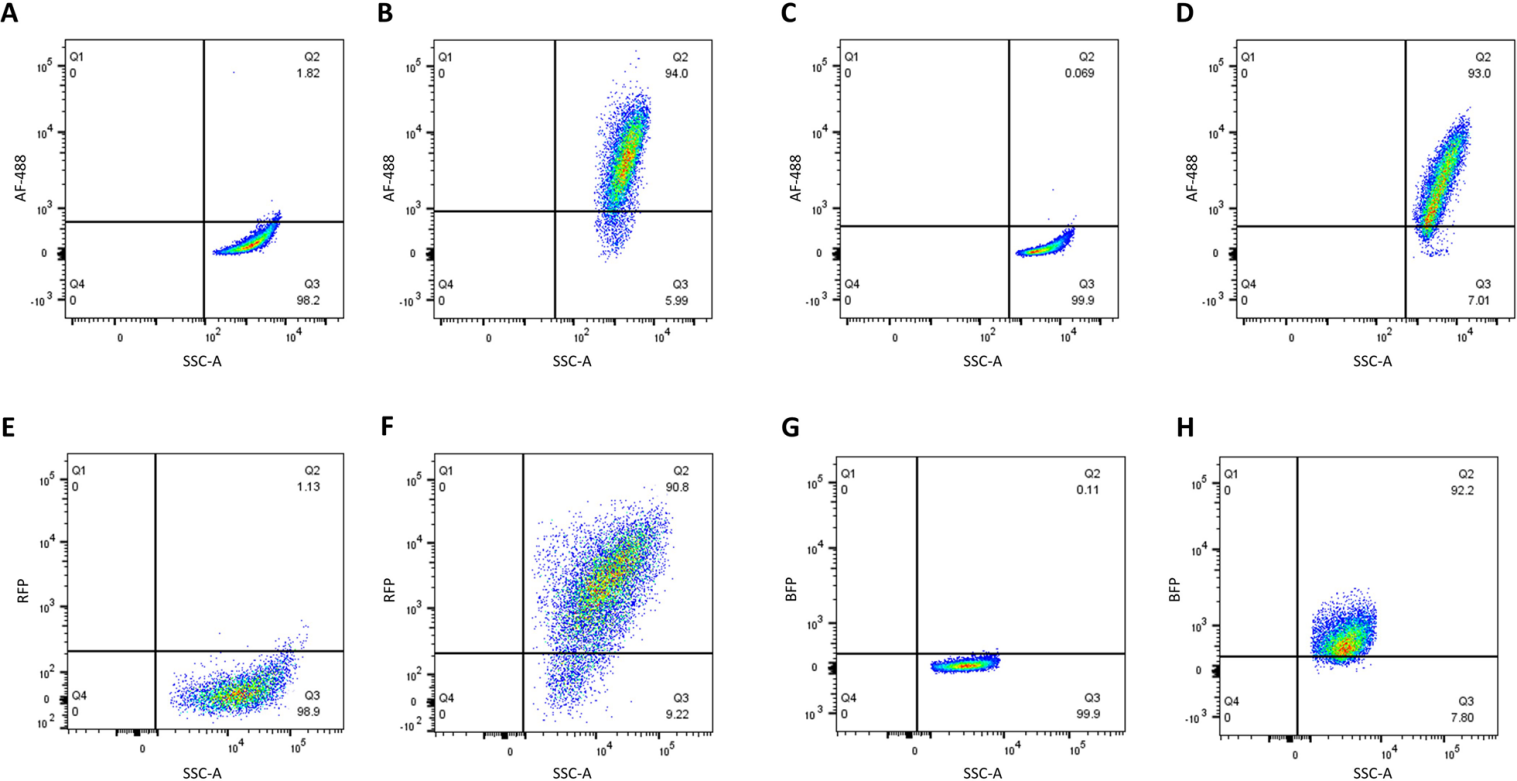
Binding specificity of nanobodies by flow cytometry. *Lactobacillus* strains were incubated with Lj94, Lc38, Lc58RFP, or Lj75BFP and analyzed by flow cytometry. *L. crispatus* EX8 cells were incubated with Dylight alone (**A**) or Dylight plus 10 µg/ml Lc38 (**B**). *L. jensenii* JV-V16 cells were incubated with Dylight alone (**C**) or Dylight plus 10 µg/ml Lj94 (**D**). *L. crispatus* 33820 cells (**E**) or incubated with 10 µg/ml Lc58 (**F**). *L. jensenii* 115-3-CHN cells (**G**) or incubated with 40 µg/ml Lj75BFP (**H**). This experiment was repeated twice with similar results

To assess the ability of these nanobodies to bind *L. crispatus* and *L. jensenii* cells in suspension, after growth in liquid media, cell preparations were probed with Lc38 or Lj94 and a secondary anti-his Alexa Fluor 488-conjugated antibody, or with Lc58RFP or Lj75BFP alone, and analyzed by flow cytometry. Nanobody binding was indicated by a positive shift in fluorescence signal intensity compared to the control (Fig. 2). A positive shift in *L. crispatus* EX8 VC07, *L. jensenii* JV-V16, *L. crispatus* 33820, and *L. jensenii* 115-3-CHN fluorescence intensity compared to the control was observed when these cells were treated with Lc38, Lj94, Lc58RFP, or Lj75BFP, respectively (Fig. 2A–H). For example, 90% of *L. crispatus* 33820 cells incubated with Lc58RFP exhibited a higher red fluorescence signal than the untreated *L. crispatus* 33820 control (Fig. 2E, F). The two *L. crispatus* nanobodies examined, Lc58 (131 AA) and Lc38 (110 AA), share 56% AA identity and bound the same subset of *L. crispatus* strains tested, both according to colony immunoblot and flow cytometry (Tables 1 and 2). This indicates that the antigen(s) recognized by these nanobodies are displayed on the surface of cells grown in broth or and detectable on colony immunoblots and that fusion of Lc58 to RFP did not impede Lc58 binding to the target cells. The two *L. jensenii* nanobodies studied, Lj75 (128 AA) and Lj94 (125 AA), shared 65% AA identity. As with the *L. crispatus* nanobodies, Lj75 and Lj94 colony immunoblot and flow cytometry binding data were consistent with each other (Tables 1 and 2), indicating that Lj75 fusion to BFP did not prevent Lj75 binding to target cells. Interestingly, Lj94 colony immunoblotting exhibited variable binding that appeared to depend on residual cells that remained attached to the filter (data not shown). In contrast, Lc58, Lc38, and Lj75 exhibited consistent binding properties in flow cytometry and immunoblotting assays. Lc58RFP and Lj75BFP fusion proteins were used in the flow cytometry binding assay because they exhibited the same binding spectra as their respective untagged proteins in the immunoblot assay and they were used in downstream experiments where the use of untagged nanobodies would not enable differentiation of cells based on detection of nanobody binding with an anti-His secondary antibody (i.e., both nanobodies have a His-tag).

**Table 2:** Flow cytometry binding specificity of nanobodies Lc58 and Lj75

Strain	Lc58	Lc38	Lj75	Lj94
*L. crispatus* EX533959 VC05	−	−	n/a	n/a
*L. crispatus* EX849587 VC07	+	+	n/a	n/a
*L. crispatus* 33820	+	+	−	n/a
*L. jensenii* 115-3-CHN	−	n/a	+	−
*L. jensenii* JV-V16	n/a	n/a	+	+
*L. jensenii* 25258	n/a	n/a	−	−

### Identification of proteins bound by nanobodies Lc58 and Lj75

**Fig. 3:**
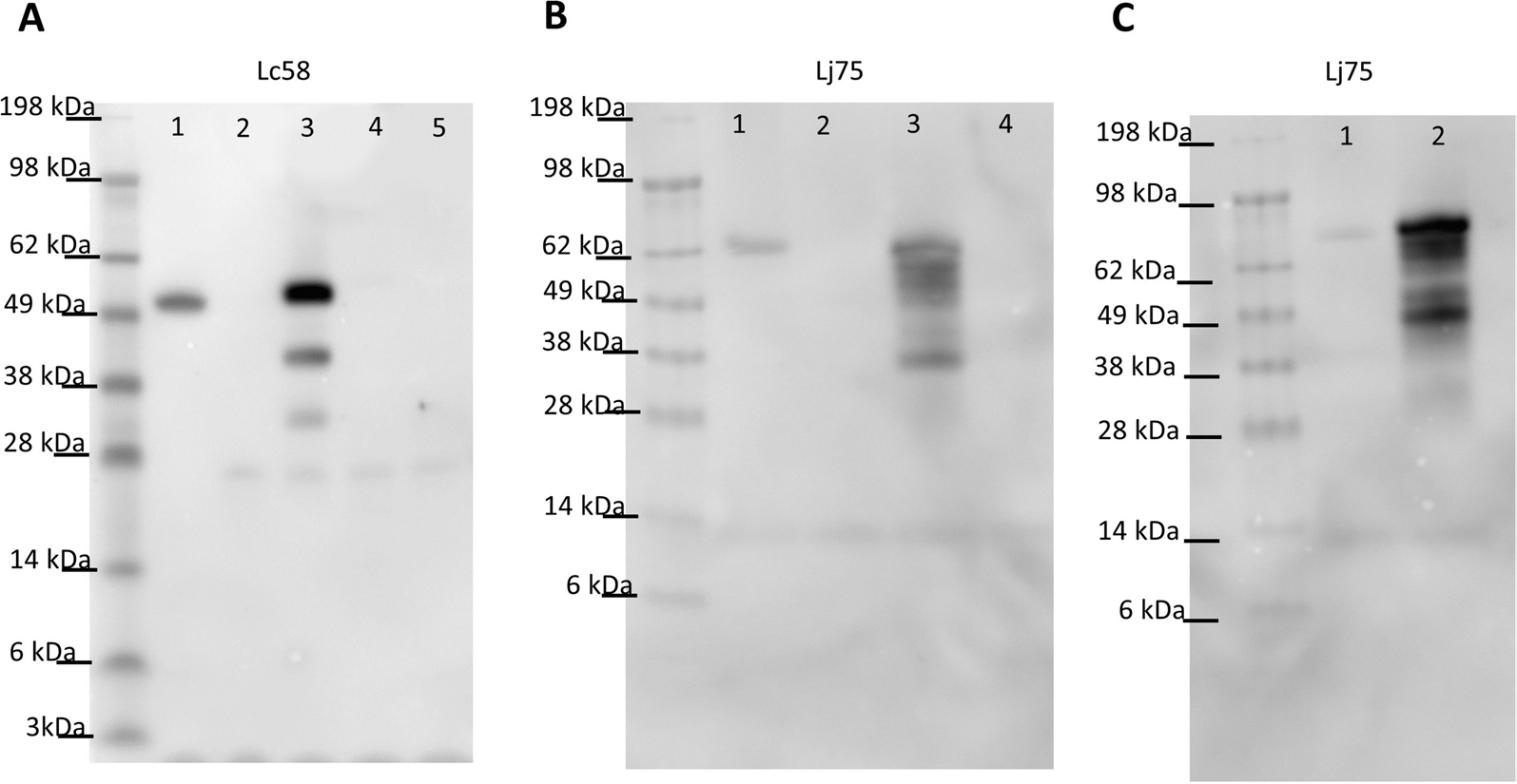
Identification of antigens bound by Lc58 and Lj75 and culture conditions under which they were detected. **A** An approximately 55-kDa band was detected in the *L. crispatus* 125-2-CHN lysate (lane 1) and in the *E. coli* lysate containing the plasmid expressing *L. crispatus* 125-2-CHN S-layer protein, EEU18441.1 (lane 3), when probed with Lc58. No bands were observed in the *E. coli* lysates from cells containing the empty vector (lane 2), the plasmid expressing Bacterial Ig-like domain protein, EEU19392.1 (lane 4), or the plasmid expressing cell separation protein, EEU18637.1 (lane 5). **B** An approximately 70-KDa band was observed in the *L. jensenii* JV-V16 lysate (lane 1) and in the *E. coli* lysate containing the plasmid expressing NlpC/P60 protein, EFH30000.1 (lane 3), when probed with Lj75. No bands were observed in the *E. coli* lysates from cells containing empty vector (lane 2) or the plasmid expressing the uncharacterized protein, EFH30544.1 (lane 4). **C** When probed with Lj75, an approximately 80-kDa band was observed in the *L. jensenii* 115-3-CHN lysate (lane 1) and in the *E. coli* lysate containing the plasmid expressing the full length NlpC/P60 protein EEX23860.1 (lane 2)

The antigens targeted by Lc58 and Lj75 were identified using mass spectrometry. Western blots of cell lysates of binding-positive *Lactobacillus* strains probed with nanobodies revealed that Lc58 bound to an approximately 50 kDa *L. crispatus* 125-2-CHN and *L. crispatus* EX8 VC07 protein (Fig. 3A lane 1 and Additional file 1: Fig. S4). Lc38 was not characterized because it bound the same set of *L. crispatus* strains as Lc58 and because it also bound a similarly sized 50 kDa target protein (data not shown). Lj75 bound to proteins of approximately 70 kDa and 80 kDa in *L. jensenii* JV-V16 and *L. jensenii* 115-3-CHN, respectively (Fig. 3B, lane 1 and C, lane 1). The antigen bound by Lj94 was not characterized because of the variable binding we observed in the immunoblot assay. Proteins within the appropriate size ranges were extracted from the gel and subjected to mass spectrometry for identification. MS analysis of *L. crispatus* 125-2-CHN proteins in the 55-kDa region revealed 4 candidate proteins that exhibited more than 2 unique peptides, a predicted signal peptide, and an annotated predicted size consistent with that predicted from the gel, 50–60 kDa in this case (Table 3). MS analysis of the *L. jensenii* JV-V16 band revealed 2 candidate epitope proteins that met our criteria (Table 2). The *L. jensenii* 115-3-CHN candidate protein (EEX23860.1) was identified by searching the *L. jensenii* 115-3-CHN protein database using the predicted AA sequence of the *L. jensenii* JV-V16 candidate, EFH30000.1, as a query (details provided in “Materials and methods”).

**Table 3:** Membrane proteins identified by LC–MS

	Lc58 candidate accession	Description	MW (kDa)
*L. crispatus* 125-2-CHN	EEU18441.1	Putative S-layer	52.6
	EEU19392.1	Bacterial Ig-like	55.8
	EEU18637.1	Cell separation protein	62.9

	Lj75 candidate accession	Description	MW (kDa)
*L. jensenii* JV-V16	EFH30544.1	Uncharacterized protein	76.8
	EFH30000.1	NlpC/P60 family protein	70.0
*L. jensenii* 115-3-CHN	EEX23860.1	NlpC/P60 family protein	38.2
	EEX23860.1_EX	NlpC/P60 family protein	80.6

Identity of the antigens bound by each nanobody was determined by probing Western blots of *E. coli* lysates expressing FLAG-tagged candidate antigens. The expression of each antigen candidate was confirmed by probing Western blots with a Monoclonal-FLAG M2 antibody (Additional file 1: Fig. S5). When candidate blots were probed with Lc58, the lane containing the S-layer protein (Fig. 3A, lane 3) exhibited a band similar in size to that seen with the *L. crispatus* 125-2-CHN lysate (Fig. 3A, lane 1). Probing of candidate blots with Lj75 resulted in bands in lanes corresponding to *E. coli* expressing NlpC/P60 family proteins EFH3000.1 and EEX23860.1 (Fig. 3B, lane 3, and Fig. 3C, lane 2). These results demonstrated that Lc58 and Lj75 bind to an S-layer protein (EEU18441.1) and an NlpC/P60 protein (EFH30000.1 and EEX23860.1_EX), respectively. Moreover, among the sequenced strains tested, the coding sequence for each antigen detected by one of these nanobodies was present within the genomes of binding strains, but not in the genomes of non-binding strains (data not shown).

### Characterization of Lj75 and Lc58 antigen accessibility

**Fig. 4:**
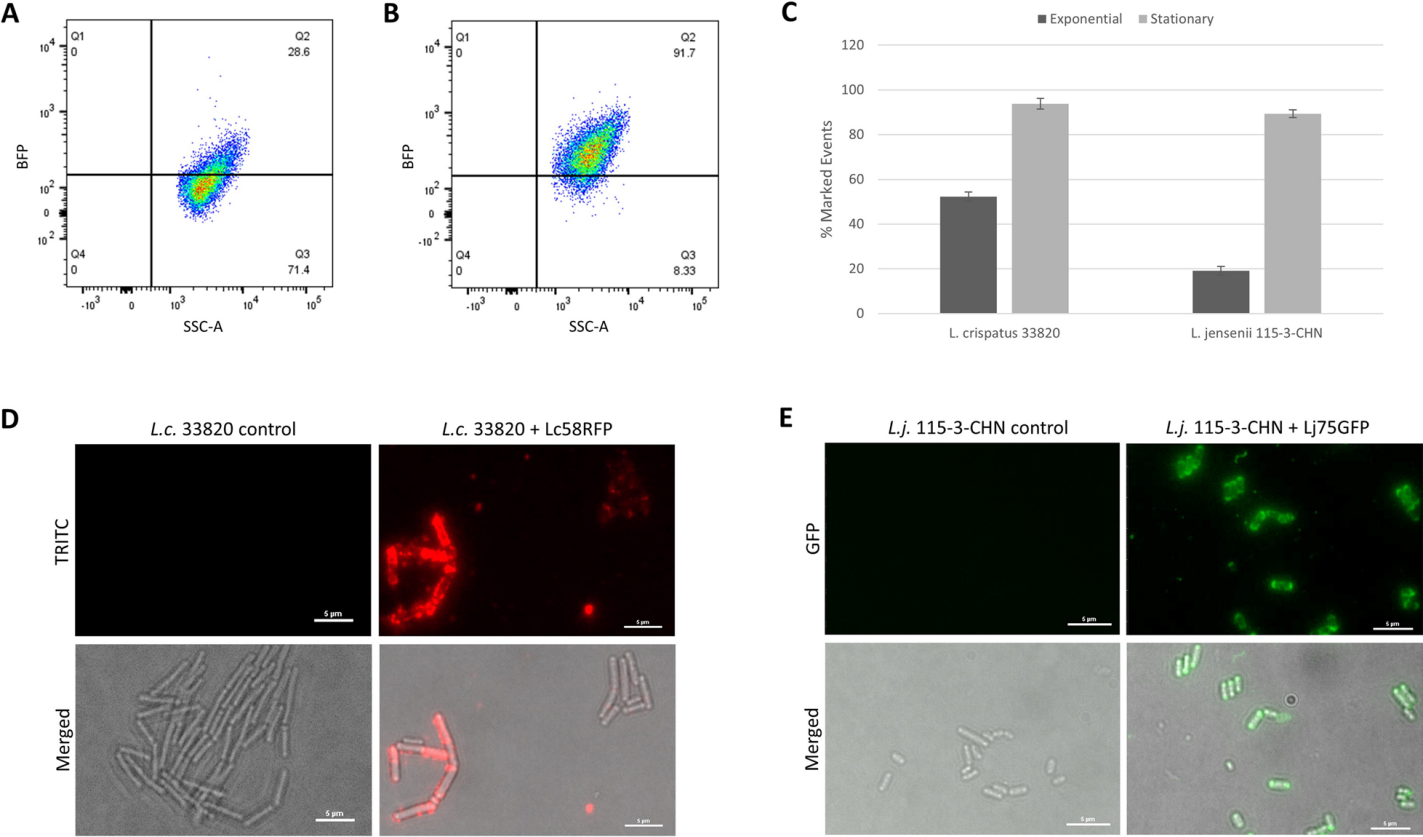
Characterization of antigen display. Binding of *Lactobacillus* by Lc58 and Lj75 was assessed at different growth phases by flow cytometry and visualized by fluorescence microscopy. **A** Exponential and **B** stationary phase *L. jensenii* 115-3-CHN cells labeled with Lj75BFP. **C** Percentage of exponential or stationary phase-grown *L. crispatus* or *L. jensenii* bound by Lc58RFP or Lj75BFP, respectively, by flow cytometry. Data points represent the mean ± standard deviation from six biological replicates in two independent experiments. **D** Unlabeled *L. crispatus* control (left) and *L. crispatus* 33820 labeled with Lc58RFP (right), shown with fluorescence only (top) and merged brightfield and fluorescence (bottom). **E** Unlabeled *L. jensenii* 115-3-CHN (left) and *L. jensenii* 115-3-CHN labeled with Lj75GFP (right) shown with fluorescence only (top) and merged brightfield and fluorescence (bottom)

The bacterial surface is dynamic and the presence and binding accessibility of PG modification enzymes, such as the NlpC/P60 family protein detected by Lj75, and S-layer proteins, such as that detected by Lc58, may vary depending on bacterial growth phase. Nearly all stationary phase *L. jensenii* cells were bound by Lj75BFP, whereas only 20% of exponential phase cells were bound (Fig. 4A–C). Similarly, Lc58RFP bound almost all stationary phase *L. crispatus* cells, whereas approximately half of exponential phase *L. crispatus* were bound (Fig. 4C). These results suggest that both the *L. crispatus* S-layer and *L. jensenii* NlpC/P60 peptidase antigen accessibility and detection is growth-phase dependent and that both proteins are most available for nanobody binding when the cells are in stationary phase. These results are consistent with the growth stage of the bacteria used to immunize llamas for isolation of nanobodies. This suggests that the cell surface characteristics of product strains under the conditions in which they will be tested should be considered when developing similar reagents, to ensure optimal binding and detection of cells under the conditions in which they will be investigated. In the case of nanobodies Lc58 and Lj75, the strains should be grown to stationary phase to ensure optimal binding and accurate strain enumeration.

To further our understanding of Lj75 and Lc58 binding characteristics, fluorescence microscopy was used to assess nanobody cellular binding patterns. Lc58RFP-bound *L. crispatus* 33820 cells varied from very bright, complete, or patchy surface decoration to faint, patchy decoration (Fig. 4D). These results are consistent with the range of fluorescence intensity observed with Lc58RFP-bound *L. crispatus* 33820 cells in flow cytometry binding assays and suggest that the S-layer antigen is not uniformly displayed or accessible on *L. crispatus* 33820 cell surfaces (Fig. 2F). The S-layer protein bound by Lc58 shares AA sequence similarity (97% coverage, 70% identity) with the *L. acidophilus* minor S-layer protein, SlpX, which is an ancillary S-layer protein thought to associate with other *L. acidophilus* S-layer proteins (Goh et al. 2009). The binding patterns observed may thus reflect the differential accessibility of the antigen for binding due to its interaction with other S-layer or S-layer associated proteins. The *L. jensenii* 115-3-CHN cell surfaces were completely decorated with Lj75GFP, with higher fluorescence intensity observed at the cell poles (Fig. 4E). Most *L. jensenii* 115-3-CHN cells were labelled and there was little variation in fluorescence intensity, which is also consistent with flow cytometry analysis (Fig. 2H). Overall these results reinforce the flow cytometry binding data and provide visualization of how Lj75BFP decorates nearly the entire surface of *L. jensenii* 115-3-CHN cells whereas Lc58RFP binding to *L. crispatus* 33820 cells is more variable.

### Fluorescent nanobody binding can differentiate *L. crispatus* and *L. jensenii* when evaluated by fluorescence microscopy

**Fig. 5:**
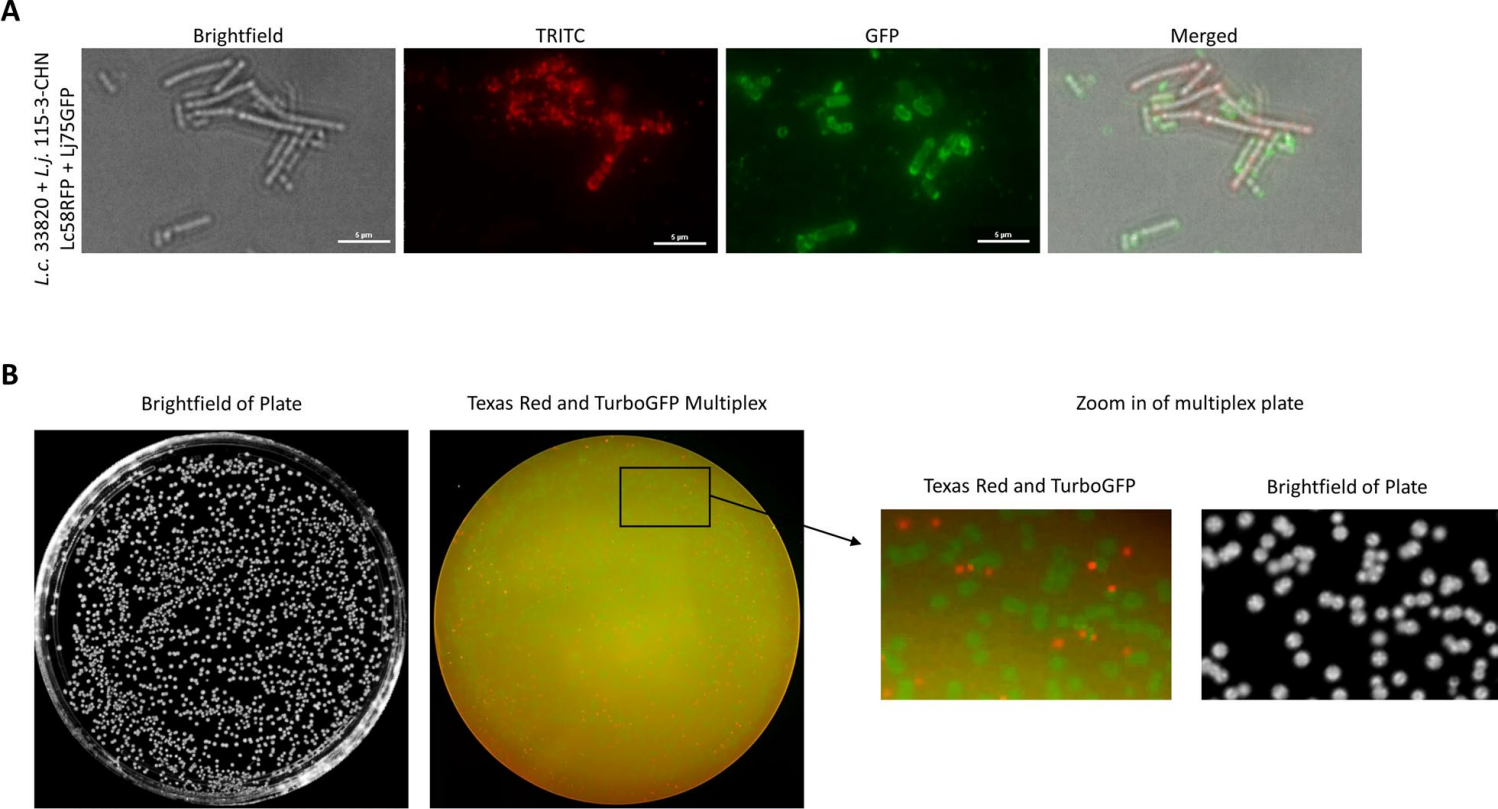
Differentiation of *L. crispatus* and *L. jensenii* cells by colony immunoblots and fluorescence microscopy. **A** *L. crispatus* 33820 and *L. jensenii* 115-3-CHN mixture incubated with Lc58RFP and Lj75GFP and imaged as indicated. The images are representative of two independent experiments. **B** Colony immunoblot of a *L. crispatus* 33280 and *L. jensenii* 115-3-CHN mixture performed as described in “Materials and methods”. An image of the plate prior to colony lift (L), a fluorescence multiplex image of the colony immunoblot (M), and an enlarged image of a portion of the blot are shown. The images are representative of three independent experiments

In-process controls, such as assessment of the presence and abundance of individual strains during multi-strain LBP fermentation, can be an important part of developing a manufacturing process to consistently manufacture a multi-strain product. Assessment of fluorescent nanobody binding by fluorescence microscopy is one approach to rapidly assess the bacterial contents of a suspension. To assess the feasibility of such an assay, *L. crispatus* 33820 and *L. jensenii* 115-3-CHN cell mixtures were treated with Lc58RFP and Lj75GFP and visualized by fluorescence microscopy. As anticipated, this analysis revealed two populations of cells: one bound by Lc58RFP, fluorescing red, and another bound by Lj75GFP, fluorescing green (Fig. 5A). The red fluorescent cells displayed similar cell size and shape as well as binding characteristics of the Lc58RFP-treated *L. crispatus* (Figs. 4D and 5A). Similarly, the green cells were similar in size, cell shape, and binding characteristics to the Lj75GFP-treated *L. jensenii* (Figs. 4E and 5A). These results show that fluorescence microscopy, together with the reagents we have created, can be used to quickly assess a bacterial mixture for the presence of different *Lactobacillus* strains.

### Differentiation of *L. crispatus* and *L. jensenii* colonies fluorescent nanobody binding in a colony immunoblot assay

LBP potency is typically determined by plating product strains to determine CFU per dose. In the case of a multi-strain product, differentiation of individual strains by colony morphology may be challenging. An alternative method for identifying colonies is colony immunoblotting. Colonies of an *L. crispatus* and *L. jensenii* mixture were transferred from an MRS plate to a nitrocellulose filter and probed with Lc58RFP and Lj75GFP. Visualization of the blots enabled the differentiation of red fluorescing *L. crispatus* colonies and green fluorescing *L. jensenii* colonies (Fig. 5B). These results show that fluorescent nanobody binding of colonies can be used to differentiate colonies using a low-tech colony immunoblot assay, making it readily applicable to the determination of viable individual cell counts in a multi-strain LBP.

### Fluorescent nanobodies can distinguish and enumerate viable *L. crispatus* from *L. jensenii* in mixture by flow cytometry

**Fig. 6:**
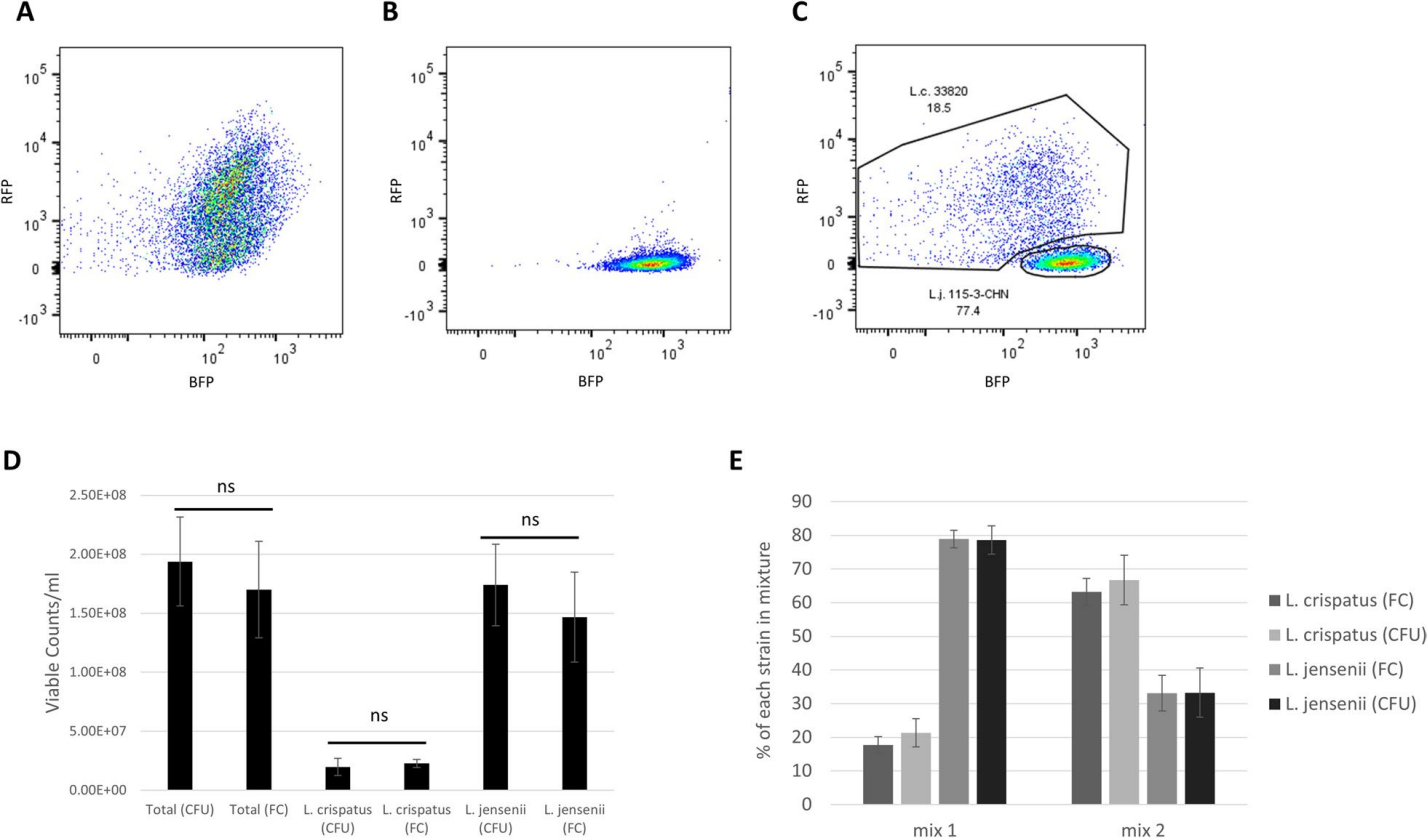
Flow cytometry analysis of a *L. crispatus* 33820 and *L. jensenii* 115-3-CHN strain mixture incubated with Lc58RFP and Lj75BFP. Dot-plots of viable *L. crispatus* 33820 control (**A**), *L. jensenii* 115-3-CHN control (**B**) and a mixture (**C**). **D** Comparison of viable counts of *L. crispatus* 33820 and *L. jensenii* 115-3-CHN cells as determined by flow cytometry (FC) and CFU. Data points represent the mean ± standard deviation from three biological replicates. **E** Comparison of the relative abundance of *L. crispatus* and *L. jensenii* in two different mixtures as measured by flow cytometry and CFU. The data points represent mean ± standard deviation from three independent experiments

Traditional methods to assess LBP potency rely on determining CFU of individual strains. In addition to potentially being unable to differentiate strains by colony morphology, traditional plate count methods may take 48–72 h and may have difficulties detecting strains at low abundance. An alternative and quicker approach to measure the viable counts of multiple bacterial strains in mixture is flow cytometry. Flow cytometric detection of fluorescent nanobody binding was used to discriminate and enumerate viable *L. crispatus* and *L. jensenii* cells in mixture. A live/dead stain was employed along with the fluorescent nanobodies to distinguish live from dead cells and ensure that only live cells were analyzed (Additional file 1: Fig. S6). Individual *L. crispatus* 33820 and *L. jensenii* 115-3-CHN preparations were incubated with both Lc58RFP and Lj75BFP to determine the dot-plot patterns of each strain incubated with both fluorescent nanobodies. The two strains exhibited distinct patterns that were used to create gates which were then applied to differentiate *L. crispatus* 33820 and *L. jensenii* 115-3-CHN cells in a mixture (Fig. 6A, B). Flow cytometry diagrams of the two-strain mixture exhibited two distinct density foci, each corresponding to the pattern observed in the *L. crispatus* 33820 or *L. jensenii* 115-3-CHN single strain control (Fig. 6A–C). Incorporation of absolute counting beads (CountBright, Invitrogen, C36950) into the fluorescent nanobody flow cytometry assay was used to allow quantitation of cell on a per volume basis. To confirm the flow cytometry enumeration results, they were compared to CFU generated using a traditional plating method coupled with colony identification by MALDI-TOF-MS. The viable counts determined by flow cytometry were not statistically different (t-test, P > 0.05) from the CFU obtained with a traditional plating method and MALDI-TOF-MS (Fig. 6D). Taken together, these results indicate that detection of nanobody binding by flow cytometry can be used to accurately assess both the relative and absolute abundance of viable cells of two *Lactobacillus* strains in mixture and provide support for the feasibility of such assays for testing of multi-strain LBPs.

*Lactobacillus crispatus* and *L. jensenii* cultures were mixed in two different volumetric proportions and analyzed by CFU and flow cytometry to determine whether flow cytometric detection of nanobody binding can accurately measure the proportion of live cells of each strain in the mixture. The mixtures were plated onto MRS agar and colonies were of each were identified by MALDI TOF and counted. Mixture 1 was composed of approximately 20% *L. crispatus* and 80% *L. jensenii* and mixture 2 is made of 70% *L. crispatus* and 30% *L. jensenii* (Fig. 6E). The mixtures were analyzed with flow cytometry using the same gating strategy as described above but without the addition of the absolute counting beads. When analyzed by flow cytometry, mixture 1 was composed of 17.7 ± 2.5% *L. crispatus* and 78.8 ± 2.6% *L. jensenii* (Fig. 6E). Flow cytometry analysis showed that mixture 2 consisted of 63.2 ± 3.9% *L. crispatus* and 33.1 ± 5.3% *L. jensenii* (Fig. 6E). In each mixture, the proportions of *L. crispatus* and *L. jensenii* colonies isolated were similar to the proportions of *L. crispatus* and *L. jensenii* identified by flow cytometry. The maximum and minimum relative abundance of each strain that could be accurately differentiated using this method was not determined. The mixtures tested here included 20% *L. crispatus* and 80% *L. jensenii* and 70% *L. crispatus* and 30% *L. jensenii*, suggesting the flow cytometry analysis is able to accurately determine the relative abundance of each strain when mixed at these proportions (Fig. 6E).

## Discussion

Nanobodies Lc58, Lc38, Lj75, and Lj94 were selected from a VHH library generated from llamas immunized with radiation-killed whole cell *L. jensenii* or *L. crispatus* preparations. All four nanobodies were found to exhibit strain-specific binding in colony immunoblot and flow cytometry assays. Furthermore, Lc58 and Lj75 nanobody binding was shown to be directed to specific surface exposed proteins. Fluorescent tagging of Lc58 and Lj75 enabled assessment of these proteins in LBP microbiological testing assays, including multiplex assays by colony immunoblots and flow cytometry. These results demonstrate that the immunization strategy allowed for the identification of manipulatable and purifiable functional nanobodies that can be utilized for microbiological testing of LBPs.

The nanobodies evaluated in this study were generated by immunizing llamas with radiation-killed whole cell preparations of S-layer producing or non-S-layer producing *Lactobacillus* cells. This mode of immunization allowed for the targeting of prominently displayed surface antigens. Use of whole cells in the subsequent panning, enrichment, and screening steps led to the isolation of nanobodies with strain-specific binding capabilities. Additionally, this unbiased immunization approach allowed for the identification of new, surface exposed, immunogenic proteins. Previous studies have used a similar inactivated whole cell immunization approach to generate strain-specific nanobodies or polyclonal antibodies (Bellais et al. 2022; Tu et al. 2016). For example, camels immunized with heat-killed *Brucella* generated nanobodies that targeted unique strain-specific antigens capable of distinguishing between the closely related organisms *Brucella* and *Yersinia* (Tu et al. 2016). An alternative, although biased, approach is to generate nanobodies or other antibodies against well-known prominent surface exposed proteins. A recent study used this approach to generate anti-S-layer polyclonal antibodies for depleting or enriching *L. acidophilus* cells in microbial consortia (Marcos-Fernández et al. 2021). The unbiased approach used herein enabled the isolation of nanobodies with strain-specific binding capabilities and led to the identification of classes of surface displayed antigens accessible to such reagents. These antigens may be exploited, in a more biased approach, for future development of LBP testing reagents and research tools to characterize the surface of *Lactobacillus* cells as well as other genera.

The *Lactobacillus* cell wall is a complex and dynamic structure and its characteristics, such as peptidoglycan structure and S-layer production, can be influenced by environmental conditions and growth phase (Kleerebezem et al. 2010; Martínez et al. 2020). Two potential applications for the nanobodies identified here are differentiation of bacteria grown on agar plates by colony immunoblots and bacteria in solution by flow cytometry. The *L. crispatus* specific nanobodies, Lc38 and Lc58, were found to bind the same subset of *L. crispatus* strains in both binding assays and to bind a ~ 50 kDa *L. crispatus* protein despite sharing only 55% AA sequence identity (data not shown). Interestingly, the *L. jensenii* nanobodies, Lj75 and Lj94, exhibited different binding spectra in the binding assays. Lj75 bound the same subset of *L. jensenii* strains in both assays whereas Lj94 only bound *L. jensenii* JV-V16. These results show that these nanobody targeted antigens are accessible for nanobody binding in both conditions, indicating that these nanobodies may be suitable for use in studies of mock multiplex colony immunoblot and flow cytometry assays. Given their suitable binding characteristics in both experimental conditions, Lj75 and Lc58 were chosen for further characterization.

The inherent requirements that were imposed during the immunization, panning, and screening process for the nanobodies reported here were that they be surface exposed, well expressed, and specific to the particular immunizing strains. Lc58 was isolated as specifically binding the S-layer producer *L. crispatus* and recognizes a strain specific S-layer protein. This finding is consistent with the known role of S-layers as prominent components of the *L. crispatus* cell surface and immunomodulatory activities of *Lactobacillus* S-layers (Goh et al. 2021; Hymes et al. 2016; Hynönen and Palva 2013; Johnson et al. 2016; Konstantinov et al. 2008; Prado Acosta et al. 2019; Sillanpää et al. 2000; Taverniti et al. 2013). Lj75 was isolated as binding to *L. jensenii* and specifically recognizes a strain-specific NlpC/P60 peptidase antigen. NlpC/P60 proteins are a broad class of cell wall hydrolases found in diverse bacterial lineages, and are known to be involved in peptidoglycan turnover, suggesting positioning near the cell surface (Bäuerl et al. 2019; Duchêne et al. 2019). The specific roles of these antigens in human-microbe interactions are largely unknown. However, if specific surface exposed proteins were shown to contribute to the desired beneficial effect(s) or mechanism of action of an LBP, such nanobody probes could potentially be used as an additional measurement of potency and/or manufacturing consistency. Finally, the antigens used to immunize the llamas are only found in a subset of strains within each *Lactobacillus* species that we tested, providing the possibility of species and strain-level differentiation.

Fluorescent labeling of nanobodies Lj75 and Lc58 by fusion to fluorescent proteins enabled characterization of target strain cell surfaces as well as the development of procedures to differentiate and/or enumerate viable *L. crispatus* and *L. jensenii* cells in mixture by fluorescence microscopy, colony immunoblots, and flow cytometry. Linking of nanobody domains to heterologous functional domains, such as fluorescent proteins, effectors, or nanobody domains is typically achieved by spacing the domains with linker regions, such as glycine-serine rich linkers and linkers derived from immunoglobulin molecules (Brinkmann and Kontermann 2017; Conrath et al. 2001). In this study, a glycine-serine rich linker (G_4_S)_3_ was used to link nanobodies Lj75 and Lc58 to fluorescent proteins and these proteins possessed both functional nanobody binding and fluorescence activity, as shown by no difference in Lc58 and Lj75 binding spectra in flow cytometry (fused protein) and colony immunoblot data (nanobody only). A similar linker has been used to create an anti-CEA and anti-CD16 bispecific nanobody for use in immunotherapy (Dong et al. 2016). While the data shown here demonstrate that the fusion proteins were fluorescent and still capable of binding the target cells, whether the fusion partner influenced fluorescence intensity of the fluorescent protein or binding affinity of the nanobody was not determined. Future work will aim to improve fluorescent nanobody production and activity (fluorescence and binding affinity) by experimenting with glycine-serine linker length, testing additional linker types, and alternative labelling approaches. One approach that might increase reagent yield while reducing total protein size and improving reagent brightness and optical characteristics is conjugation of nanobodies to small molecule fluorophores via Sortase A activity (Massa et al. 2016).

Determination of the potency of multi-strain LBP products poses a significant challenge as it should allow for the quantitation of viable organisms of each component strain. This can be difficult using conventional techniques because differentiating the strains by colony morphology or selective/indicator media may not be possible. Molecular methods to evaluate potency of probiotic product powders and suspensions by sequencing techniques and PCR assays have been described (Hansen et al. 2018; Kiefer et al. 2020; Morovic et al. 2016; Patro et al. 2016). Flow cytometry has also been used to detect and enumera*te Lactobacill*us and *Bifidobacteri*um probiotic product strains with specific fluorescently-labelled polyclonal antibodies (Chiron et al. 2018). Here we investigated the utility of fluorescent nanobody probes in evaluating a *Lactobacillus* strain mixture by colony immunoblots and flow cytometry. Detection of nanobody binding by each technique was sufficient to enumerate *L. jensenii* and *L. crispatus* colonies grown on agar plates by colony immunoblots and individual viable cells of each strain in mixture by flow cytometry. The present study builds on previous work in this space by developing fluorescent nanobody reagents that can be easily and reproducibly purified and demonstrated their utility in flow cytometry and colony immunoblot assays. Additionally, the fluorescent nanobodies enabled the detection of strains in a single binding step and did not require secondary reagents or additional steps to detect reagent binding.

The work presented here has demonstrated that fluorescent nanobodies can be useful tools to differentiate and enumerate *Lactobacillus* strains in mixture by flow cytometry or by identification of colonies grown on agar plates. As such, it is a demonstration of proof of concept for using two reagents to identify two different bacterial species. An obvious question relates to how far this approach can be pushed to identify larger numbers of different strains in more complex LBPs. For this there are two limiting factors, the multiplexing capability of the instrument and the availability of suitable reagents. The multiplexing capabilities depend on the fluorophores used as well as the ability of the instrument to limit fluorophore spectral overlap. The colony immunoblot imager and traditional flow cytometers, such as those utilized in this study, are limited by the laser and filter combinations. For example, the immunoblot imager instrument used here is potentially capable of visualizing four colors whereas the flow cytometer used can potentially distinguish 15. This suggests that the immunoblot and flow cytometry approach may be capable of assessing simpler (< 4 strains) and more complex multi-strain products (< 15 strains), respectively. It should be noted, however, that the technology and abilities of flow cytometry instruments in this area is rapidly evolving, and a greater number of channels will almost certainly be available in the near future. The other scope-determining factor is the availability of reagents. Fortunately, there are ample sources for such binding moieties, apart from nanobodies and other antibody-based reagents such as conventional polyclonal or monoclonal antibodies. Many of these can be derived from bacteriophages, which have evolved alongside their host bacteria to be capable of specific recognition. Examples of these are receptor binding proteins, phage lysin cell wall binding domains, and carbohydrate binding modules found in other structural proteins of phage particles (Dams et al. 2019; Dieterle et al. 2017; Hayes et al. 2018; Loessner et al. 2002; Schmelcher et al. 2010). Future work will focus on the characterization of such domains to create additional reagents to extend the value of the approach reported here.

### Supplementary Information

**Additional file 1:Table S1.** Bacterial strains, plasmids, and primers used in this study. **Figure S1.** ELISA results of the interaction between different lactobacilli and secreted nanobodies. (A) Lc58 and Lc38 nanobody interaction between target *L. crispatus *antigen (strains 33820 and 33197) and control lactobacilli antigens. (B) Lj94 and Lj75 nanobody interaction between target *L. jensenii *JV-V16 antigen and control lactobacilli antigens. Secreted nanobody concentrations were evaluated using Octet (Sartorius) and the preparations were diluted to 1 μg/ml for experiments. **Figure S2.** SDS PAGE gels showing purified nanobodies and fluorescently tagged nanobodies. Proteins were loaded on NuPAGE 4–12% BisTris gels and stained with Coomasie Blue. The expected molecular mass of each protein and the lane in which the purified protein was run is indicated in the boxes below the gels. Molecular weight markers are identified on the left. Please note that under boiling SDS conditions, TagRFP is known to fragment. The additional bands observed in (B) lane C are likely due to the fragmentation of sample preparation for SDS PAGE. **Figure S3.** *L. jensenii *115-3-CHN Lj75 antigen identification. (A) AA sequence analysis of (1) the originally annotated AA sequence of *L. jensenii *115-3-CHN antigen (EEX23860.1), (2) the confirmed *L. jensenii *JV-V16 Lj75 antigen AA sequence, and (3) the extended *L. jensenii *115-3-CHN Lj75 antigen AA sequence. Green above sequence analysis indicates 100% AA sequence identity. (B) Depiction of unique peptide hits along the AA sequence of the corrected *L. jensenii *115-3-CHN antigen sequence. Green indicates where in the AA sequence the unique peptides match. **Figure S4.** *L. crispatus *strain lysate western blots with Lc58. *L. crispatus *EX8 VC07 (Lane 1), *L. crispatus *125-2-CHN (Lane 2), or *L. jensenii *25258 (lane 3) lysates were probed with Lc58. Lc58 binding was detected with an anti-his HRP conjugated secondary antibody. **Figure S5.** Detection of nanobody target candidate expression by western blot with HRP conjugated anti-FLAG antibody probing. (A) Lc58 target candidates; Lane 1, *L. crispatus *125-2-CHN lysate; Lane 2, empty vector; Lane 3, S-layer (EEU18441.1) ; Lane 4, Bacterial Ig-domain protein (EEU19392.1) ; Lane 5, Cell separation protein (EEU18637.1). (B) Lj75 candidates; Lane 1, *L. jensenii *JV-V16; Lane 2, empty vector; Lane 3, NlPC/P60 family protein (EFH30000.1); Lane 4, Hypothetical protein (EFH30544.1).**Figure S6.** Use of SYTOX Green Ready Flow reagent to distinguish live from dead cells. SYTOX (ThermoFisher) is a cell impermeant nucleic acid stain that enters cells with damaged membranes and binds nucleic acids. (A) Untreated and unstained *L. crispatus *33820, (B) Untreated *L. crispatus *33820 solution (prepared same as flow cytometry samples), and (C) Isopropyl alcohol treated (70%, 25 min)*L. crispatus *33820. (D) Untreated and unstained *L. jensenii *115-3-CHN, (B) Untreated *L. jensenii *115-3-CHN solution (prepared same as flow cytometry samples), and (C) Isopropyl alcohol treated (70%, 25 min.) *L. jensenii *115-3-CHN. Please note that GFP and AlexaFluor use same laser and filter settings on the flow cytometer used in this assay.

## Data Availability

Data Generated and/or analyzed as part of this study are available upon reasonable request from the corresponding author.
