# Supplementary material for: Nanobodies as potential tools for microbiological testing of live biotherapeutic products
Source: AMB Express. 2024 Jan 20;14:9. doi: 10.1186/s13568-023-01659-z (PMC10799837; doi:10.1186/s13568-023-01659-z)
Supplement: Supplementary file 1 — Additional file 1:Table S1. Bacterial strains, plasmids, and primers used in this study. Figure S1. ELISA results of the interaction between different lactobacilli and secreted nanobodies. (A) Lc58 and Lc38 nanobody interaction between target L. crispatus antigen (strains 33820 and 33197) and control lactobacilli antigens. (B) Lj94 and Lj75 nanobody interaction between target L. jensenii JV-V16 antigen and control lactobacilli antigens. Secreted nanobody concentrations were evaluated using Octet (Sartorius) and the preparations were diluted to 1 μg/ml for experiments. Figure S2. SDS PAGE gels showing purified nanobodies and fluorescently tagged nanobodies. Proteins were loaded on NuPAGE 4–12% BisTris gels and stained with Coomasie Blue. The expected molecular mass of each protein and the lane in which the purified protein was run is indicated in the boxes below the gels. Molecular weight markers are identified on the left. Please note that under boiling SDS conditions, TagRFP is known to fragment. The additional bands observed in (B) lane C are likely due to the fragmentation of sample preparation for SDS PAGE. Figure S3. L. jensenii 115-3-CHN Lj75 antigen identification. (A) AA sequence analysis of (1) the originally annotated AA sequence of L. jensenii 115-3-CHN antigen (EEX23860.1), (2) the confirmed L. jensenii JV-V16 Lj75 antigen AA sequence, and (3) the extended L. jensenii 115-3-CHN Lj75 antigen AA sequence. Green above sequence analysis indicates 100% AA sequence identity. (B) Depiction of unique peptide hits along the AA sequence of the corrected L. jensenii 115-3-CHN antigen sequence. Green indicates where in the AA sequence the unique peptides match. Figure S4. L. crispatus strain lysate western blots with Lc58. L. crispatus EX8 VC07 (Lane 1), L. crispatus 125-2-CHN (Lane 2), or L. jensenii 25258 (lane 3) lysates were probed with Lc58. Lc58 binding was detected with an anti-his HRP conjugated secondary antibody. Figure S5. Detection of nanobody target can [file 13568_2023_1659_MOESM1_ESM.pdf]

Journal Name: AMB Express

Manuscript title: Nanobodies as Potential Tools for Microbiological Testing of Live Biotherapeutic Products

Authors: Robert J. Dorosky, Jeremy E. Schreier, Stephanie L. Lola, Rosa L. Sava, Michael P. Coryell, Adovi Akue, Mark Kukuruga, Paul E. Carlson, Jr., Sheila M. Dreher-Lesnick, and Scott Stibitz

Corresponding Author: Robert Dorosky, Robert.Dorosky@fda.hhs.gov, 570-212-1559

Table S1. Bacterial strains, plasmids, and primers used in this study.

Strain	Description	Reference or Source
Escherichia coli BL21 (DE3)	*E. coli* strain suitable for protein expression and purification	Novagen
Escherichia coli Shuffle T7	*E. coli* K12 strain engineered for improved expression and correct folding of proteins with disulfide bonds	New England Biolabs
Lactobacillus crispatus 125-2-CHN	Vaginal isolate from a healthy woman	BEI
Lactobacillus crispatus EX533959 VC04	Vaginal isolate from a healthy woman	BEI
Lactobacillus crispatus EX533959 VC05	Vaginal isolate from a healthy woman	BEI
Lactobacillus crispatus EX533959 VC06	Vaginal isolate from a healthy woman	BEI
Lactobacillus crispatus EX533959 VC07	Vaginal isolate from a healthy woman	BEI
Lactobacillus crispatus EX849587 VC01	Vaginal isolate from a healthy woman	BEI
Lactobacillus crispatus EX849587 VC02	Vaginal isolate from a healthy woman	BEI

Lactobacillus crispatus EX849587 VC04	Vaginal isolate from a healthy woman	BEI
Lactobacillus crispatus EX849587 VC07	Vaginal isolate from a healthy woman	BEI
Lactobacillus crispatus 33820	Eye isolate	ATCC
Lactobacillus crispatus 33197	Urine isolate	ATCC
Lactobacillus gasseri MV-22	Vaginal isolate from a healthy woman	BEI
Lactobacillus jensenii 115-3-CHN	Vaginal isolate from a healthy woman	BEI
Lactobacillus jensenii 25258	Human female isolate	ATCC
Lactobacillus jensenii JV-V16	Human Isolate	BEI
Lactiplantibacillus plantarum (V)	Probiotic Product Isolate	(Dreher-Lesnick et al. 2015)
Lacticaseibacillus rhamnosus (D)	Probiotic Product Isolate	(Dreher-Lesnick et al. 2015)
Lactobacillus acidophilus (V)	Probiotic Product Isolate	(Dreher-Lesnick et al. 2015)
Lacticaseibacillus paracasei (V)	Probiotic Product Isolate	(Dreher-Lesnick et al. 2015)
Lactobacillus gasseri JV-V03	female Urogenital tract isolate	BEI
Limosilactobacillus reuteri CF48-3A	Feces of healthy child	BEI
**Plasmid**	**Description**	**Source / Accession Number**

pET-22b (+)	Expression vector	Sigma-Aldrich
pJS1	pET22b (+) containing EEU18441.1 coding sequence	This Study<br>Accession: OR362328
pJS2	pET22b (+) containing EEU19392.1 coding sequence	This Study<br>Accession: OR362329
pJS3	pET22b (+) containing EEU18637.1 coding sequence	This Study<br>Accession: OR362330
pRD312	pET22b (+) containing EFH30544.1 coding sequence	This Study<br>Accession: OR362331
pRD327	pET22b (+) containing EFH30000.1 coding sequence	This Study<br>Accession: OR362332
pRD340	pET22b (+) containing EEX23860.1 coding sequence	This Study<br>Accession: OR362333
pRD314	pET22b (+) containing Lc58-TagRFP fusion coding sequence	This Study<br>Accession: OR295639
pRD309	pET22b (+) containing Lj75-TagBFP fusion coding sequence	This Study<br>Accession: OR295640
pRD446	pET22b (+) containing Lj75-TagGFP fusion coding sequence	This Study<br>Accession: OR295641
**Nanobodies**	**Description**	**Source/ Accession Number**
Lj75	*Lactobacillus jensenii* strain specific nanobody	This Study<br>Accession:

		OR295635
Lj94	*Lactobacillus jensenii* strain specific nanobody	This Study<br>Accession: OR295636
Lc38	*Lactobacillus crispatus* strain specific nanobody	This Study<br>Accession: OR295637
Lc58	*Lactobacillus crispatus* strain specific nanobody	This Study<br>Accession: OR295638
**Primers**	**Sequence 5' to 3'**	**Source**
SDL169 S-layer F	AATATTCTAGACTTTAAGAAGGAGATATA<br>CAGAAAGGAAAGGCCACA	This Study
SDL194 S- layer_FLAG R	TATGCGGCCGCTCACTTGTCATCGTCATC<br>CTTATAATCATAGAAGTTTACAGCCTT	This Study
SDL170 Ig 1 F	AATATTCTAGACTTTAAGAAGGAGATAT<br>ACGTGATTTTTTCATATGGGAGG	This Study
SDL195 Ig 1_FLAG_ R	TATGCGGCCGCTCACTTGTCATCGTCATC<br>CTTATAATCTTTAGTGCTTGTAGTCAT	This Study
SDL172 Sep F	AATATTCTAGACTTTAAGAAGGAGATATA<br>AAGTTTAGTAAGAATGAGG	This Study
SDL196 Sep R_FLAG_R	TATGCGGCCGCTCA CTTGTCATCGTCATCC<br>TTATAATCACAATAGCAGCATCCTTC	This Study

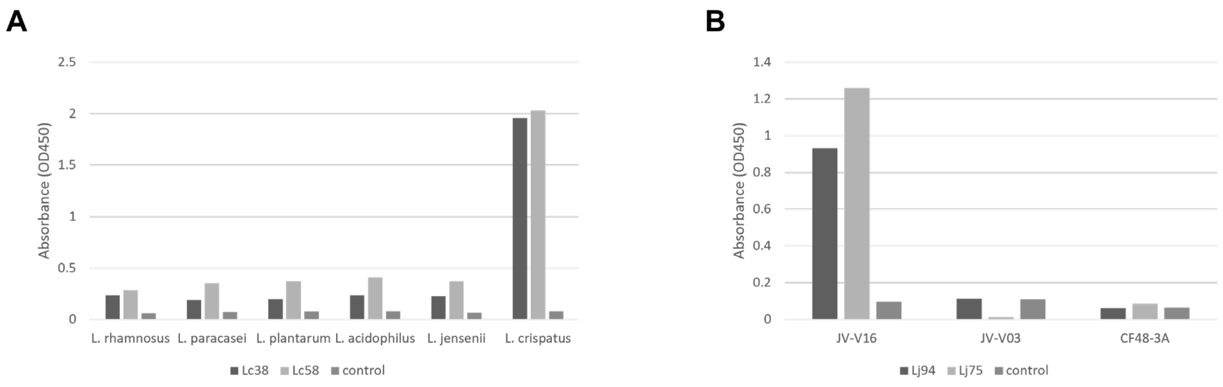

Figure S1. ELISA results of the interaction between different lactobacilli and secreted nanobodies. (A) Lc58 and Lc38 nanobody interaction between target *L. crispatus* antigen (strains 33820 and 33197) and control lactobacilli antigens. (B) Lj94 and Lj75 nanobody interaction between target *L. jensenii* JV-V16 antigen and control lactobacilli antigens. Secreted nanobody concentrations were evaluated using Octet (Sartorius) and the preparations were diluted to 1µg/ml for experiments.

A

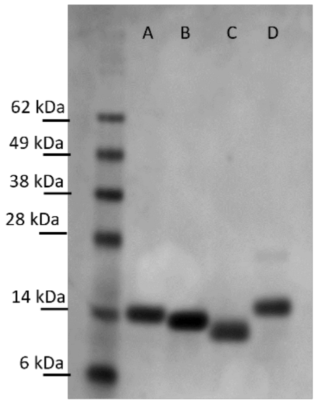

Lane	Protein	kDa
A	Lj75	14.8
B	Lj94	14.5
C	Lc38	12.9
D	Lc58	15.2

B

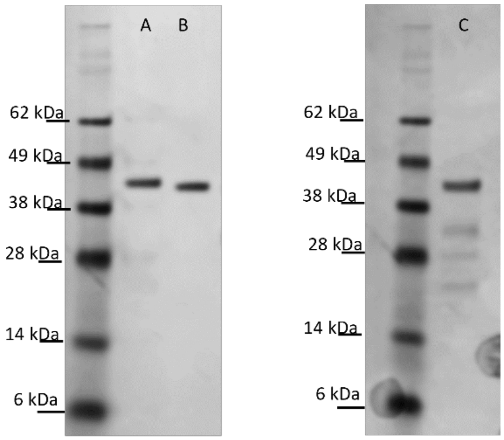

Lane	Protein	kDa
A	Lj75TagGFP	42.6
B	Lj75TagBFP	42.1
C	Lc58TagRFP	40.1

Figure S2. SDS PAGE gels showing purified nanobodies and fluorescently tagged nanobodies. Proteins were loaded on NuPAGE 4-12% BisTris gels and stained with Coomasie Blue. The expected molecular mass of each protein and the lane in which the purified protein was run is indicated in the boxes below the gels. Molecular weight markers are identified on the left. Please note that under boiling SDS conditions, TagRFP is known to fragment. The additional bands observed in (B) lane C are likely due to the fragmentation of sample preparation for SDS PAGE.

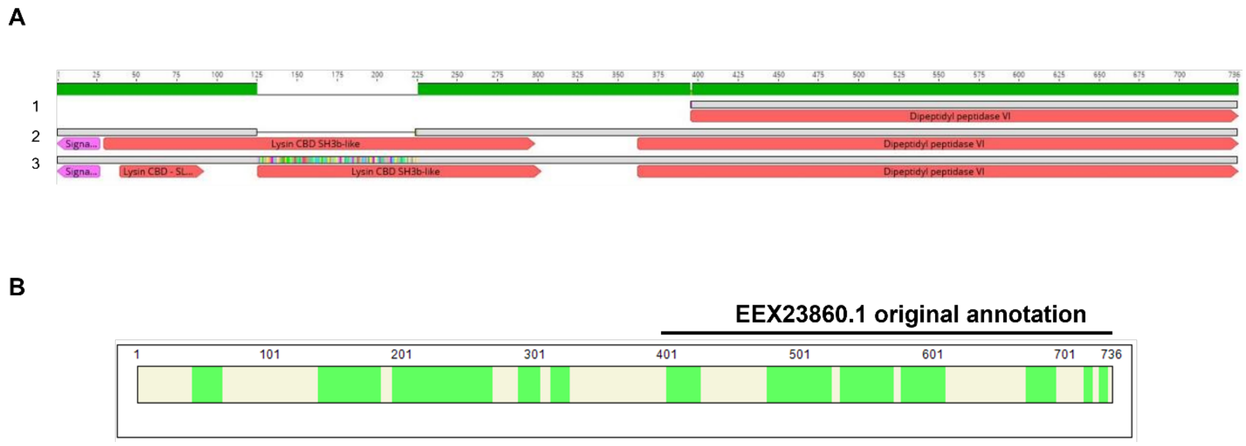

Figure S3. ***L. jensenii*** **115-3-CHN Lj75 antigen identification.** (A) AA sequence analysis of (1) the originally annotated AA sequence of *L. jensenii* 115-3-CHN antigen (EEX23860.1), (2) the confirmed *L. jensenii* JV-V16 Lj75 antigen AA sequence, and (3) the extended *L. jensenii* 115-3-CHN Lj75 antigen AA sequence. Green above sequence analysis indicates 100% AA sequence identity. (B) Depiction of unique peptide hits along the AA sequence of the corrected *L. jensenii* 115-3-CHN antigen sequence. Green indicates where in the AA sequence the unique peptides match.

Protein domains were identified by analyzing each sequence with HHpred using standard databases. (https://toolkit.tuebingen.mpg.de/tools/hhpred) and sequence analysis was performed with Geneious Prime (https://www.geneious.com/prime/).

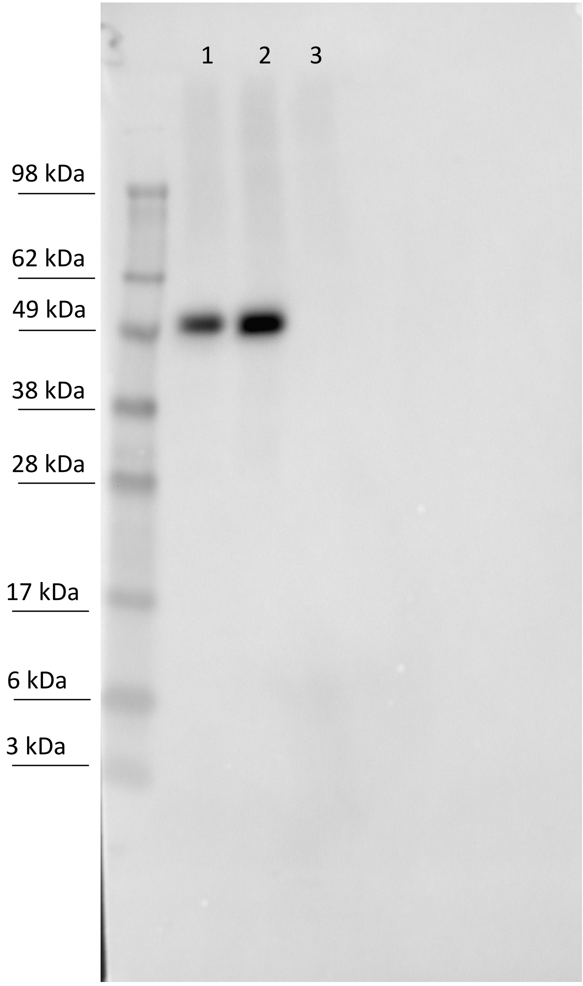

Figure S4. ***L. crispatus*** **strain lysate western blots with Lc58.** *L. crispatus* EX8 VC07 (Lane 1), *L. crispatus* 125-2-CHN (Lane 2), or *L. jensenii* 25258 (lane 3) lysates were probed with Lc58. Lc58 binding was detected with an anti-his HRP conjugated secondary antibody.

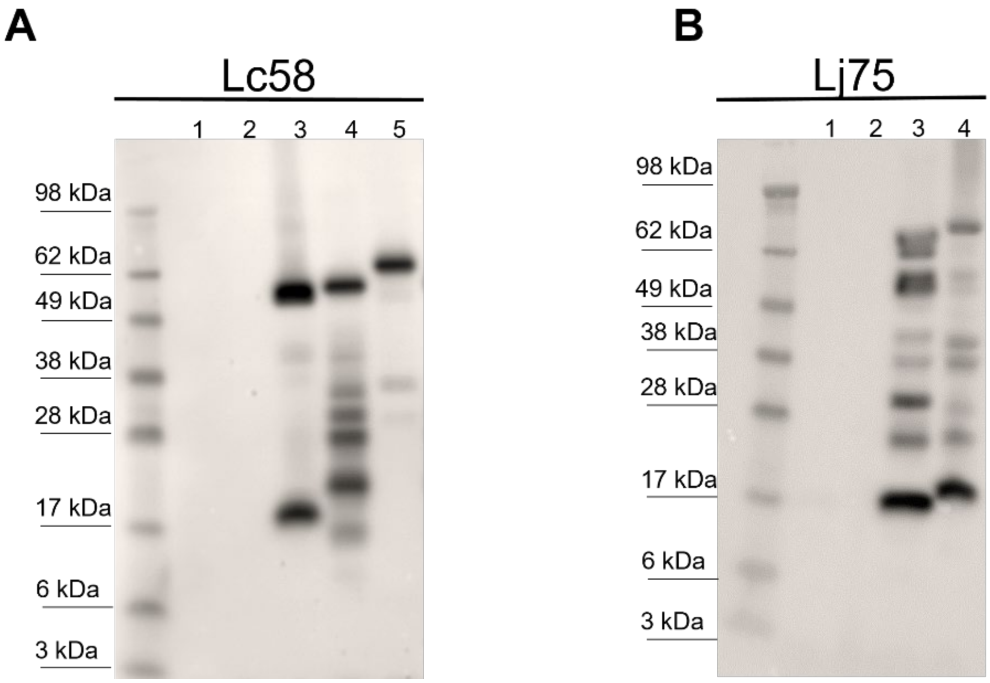

Figure S5. Detection of nanobody target candidate expression by western blot with HRP conjugated anti-FLAG antibody probing. (A) Lc58 target candidates; Lane 1, *L. crispatus* 125-2-CHN lysate; Lane 2, empty vector; Lane 3, S-layer (EEU18441.1) ; Lane 4, Bacterial Ig-domain protein (EEU19392.1) ; Lane 5, Cell separation protein (EEU18637.1). (B) Lj75 candidates; Lane 1, *L. jensenii* JV-V16; Lane 2, empty vector; Lane 3, NlPC/P60 family protein (EFH30000.1); Lane 4, Hypothetical protein (EFH30544.1).

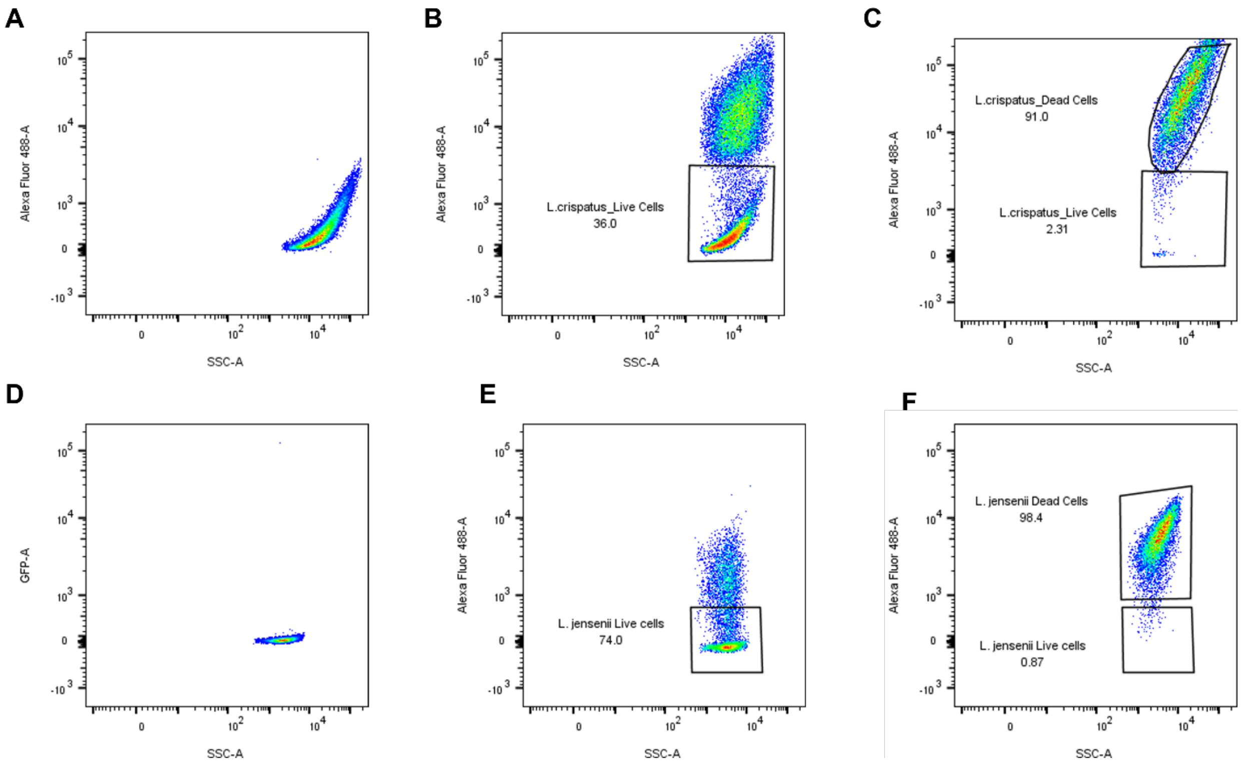

Figure S6. Use of SYTOX Green Ready Flow reagent to distinguish live from dead cells. SYTOX (ThermoFisher) is a cell impermeant nucleic acid stain that enters cells with damaged membranes and binds nucleic acids. (A) Untreated and unstained *L. crispatus* 33820, (B) Untreated *L. crispatus* 33820 solution (prepared same as flow cytometry samples), and (C) Isopropyl alcohol treated (70%, 25 min.) *L. crispatus* 33820. (D) Untreated and unstained *L. jensenii* 115-3-CHN, (B) Untreated *L. jensenii* 115-3-CHN solution (prepared same as flow cytometry samples), and (C) Isopropyl alcohol treated (70%, 25 min.) *L. jensenii* 115-3-CHN. Please note that GFP and AlexaFluor use same laser and filter settings on the flow cytometer used in this assay.

References

Dreher-Lesnick SM, Schreier JE, Stibitz S (2015) Development of phage lysin LysA2 for use in improved purity assays for live biotherapeutic products. Viruses 7(12):6675-6688
